# Acupuncture and Lifestyle Myopia in Primary School Children—Results from a Transcontinental Pilot Study Performed in Comparison to Moxibustion

**DOI:** 10.3390/medicines5030095

**Published:** 2018-08-31

**Authors:** Xiaojuan Shang, Luquan Chen, Gerhard Litscher, Yanxia Sun, Chuxiong Pan, Cun-Zhi Liu, Daniela Litscher, Lu Wang

**Affiliations:** 1 Department of Traditional Chinese Medicine, Beijing Tongren Hospital, Capital Medical University, Beijing 100730, China; joy_shang@hotmail.com (X.S.); lu.wang@medunigraz.at (L.W.); 2 TCM Research Center Graz, Research Unit of Biomedical Engineering in Anesthesia and Intensive Care Medicine, and Research Unit for Complementary and Integrative Laser Medicine, Medical University of Graz, 8036 Graz, Austria; daniela.litscher@medunigraz.at; 3 Department of Anesthesiology, Beijing Tongren Hospital, Capital Medical University, Beijing 100730, China; sun00017@gmail.com (Y.S.); pandedao@126.com (C.P.); 4 Department of Acupuncture and Moxibustion, Dongfang Hospital Affiliated to Beijing University of Chinese Medicine, Beijing 100078, China; lcz623780@126.com

**Keywords:** acupuncture, moxibustion, evidence-based complementary medicine, myopia, primary school children, lifestyle, computer, eye diseases

## Abstract

**Background:** Lifestyle risks for myopia are well known and the disease has become a major global public health issue worldwide. There is a relation between reading, writing, and computer work and the development of myopia. **Methods:** Within this prospective pilot study in 44 patients aged between 6 and 12 years with myopia we compared possible treatment effects of acupuncture or moxibustion. The diopters of the right and left eye were evaluated before and after the two treatment methods. **Results:** Myopia was improved in 14 eyes of 13 patients (15.9%) within both complementary methods. Using acupuncture an improvement was observed in seven eyes from six patients out of 22 patients and a similar result (improvement in seven eyes from seven patients out of 22 patients) was noticed in the moxibustion group. The extent of improvement was better in the acupuncture group (p = 0.008 s., comparison before and after treatment); however, group analysis between acupuncture and moxibustion revealed no significant difference. **Conclusions:** Possible therapeutic aspects with the help of evidence-based complementary methods like acupuncture or moxibustion have not yet been investigated adequately in myopic patients. Our study showed that both acupuncture and moxibustion can improve myopia of young patients. Acupuncture seems to be more effective than moxibustion in treating myopia, however group analysis did not prove this trend. Therefore, further Big data studies are necessary to confirm or refute the preliminary results.

## 1. Introduction

Myopia has become a major global public health issue worldwide. Lifestyle risk factors are well known and summarized in recent publications [1,2,3]. It has been shown that there is a relation between reading and writing from a short distance and the development of myopia. Computer work is also responsible for this relationship [4,5,6,7].

The constantly increasing number of eye diseases, as a result of too intensive personal computer work, increases the need for adequate treatment methods. In this context evidence-based complementary methods like acupuncture and/or moxibustion could be potential starting points for early intervention of myopia, which is defined as more than equal to −0.50 diopter (D) [3].

The purpose of this study was to investigate complementary medical methods (acupuncture and moxibustion) in school children with mild or moderate myopia because conventional medical therapies do not show sufficient improvements [1,2,3]. Acupuncture and moxibustion are among the most important methods used clinically in myopia in school age in China. Since this is an invasive method (needle acupuncture) on the one hand and a non-invasive procedure (moxibustion) on the other hand, it is obvious to compare both methods in one study. The aim is also to find out whether the methods differ significantly in terms of a possible improvement of myopia or not.

Within this transcontinental (Asia-Europe) prospective study in 44 children with low (<−3.0 D) and medium (between −3.0 D and −6.0 D) myopia [3] we compared possible treatment effects of acupuncture or moxibustion for the first time. The measurements were performed at the Tongren Eye Hospital affiliated to Capital Medical University in Beijing, China and the analysis was performed at the Traditional Chinese Medicine (TCM) Research Center at the Medical University of Graz, Austria. 

## 2. Materials and Methods

### 2.1. Patients

A total of 44 patients aged between 6 and 12 years, with a mean age ± SD of 9.3 ± 1.4 years (27 female, 17 male) were treated either with acupuncture (group A) or with moxibustion (group B). The children suffer from mild-to-moderate myopia (−0.5 D to −4.25 D), and their lens-corrected vision was 100%. The average stature of the 44 subjects was 137.7 ± 9.4 cm (115–157 cm), and the average body weight was 33.2 ± 7.9 kg (21–53 kg). No person was under the influence of drugs. The treatments (needle acupuncture or moxibustion) were approved by the local ethics committee of the Tongren Hospital for treatment and research (EPU 5/2017) and performed in accordance with the recommendations of the Helsinki Declaration of the World Medical Association. Informed consent has been obtained from at least one of their parents.

Acupuncture treatment was used in group A, moxibustion in group B. Each group consisted of 22 patients. Age, sex, and basic demographic data are shown in Table 1.

Randomization to one of the groups A or B has been done by an independent employee (medical doctor) from the Tongren Hospital using an envelope (group A, group B). The duration of one treatment (acupuncture or moxibustion) was 20 min. Altogether completion of 10 sessions was planned (~2 sessions per week) however not all participants could finish the examinations (compare treatment sessions in Table 1).

The following exclusion criteria were applied: (i) Less than −0.5 diopters; (ii) other eye diseases/disorders affecting visual acuity; (iii) secondary eye diseases (e.g., following diabetes); (iv) cardiologic, neurologic, nephrologic, hepatologic, hematologic, or psychiatric disorders; (v) chronic diseases requiring medication that must not be interrupted.

### 2.2. Treatment Methods

#### 2.2.1. Acupuncture

**Figure 1:**
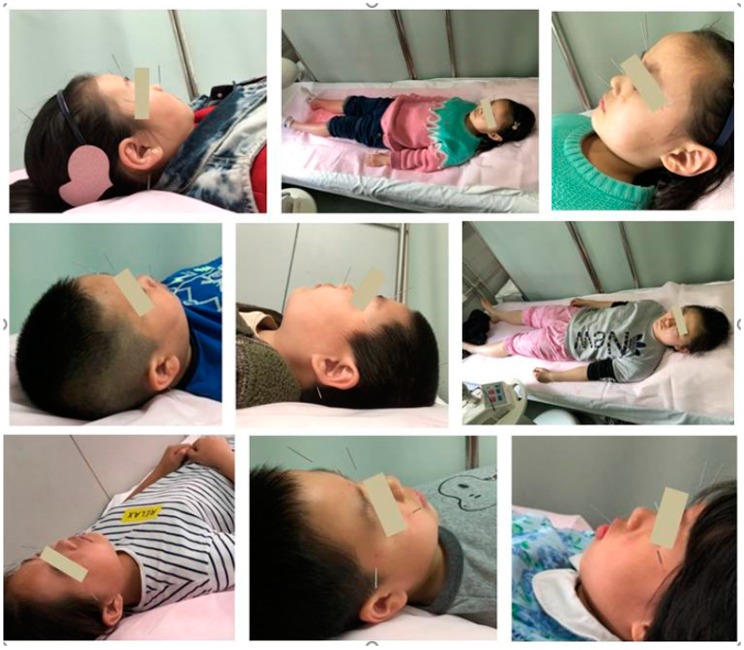
Acupuncture treatment in 22 young patients with myopia (group A).

**Figure 2:**
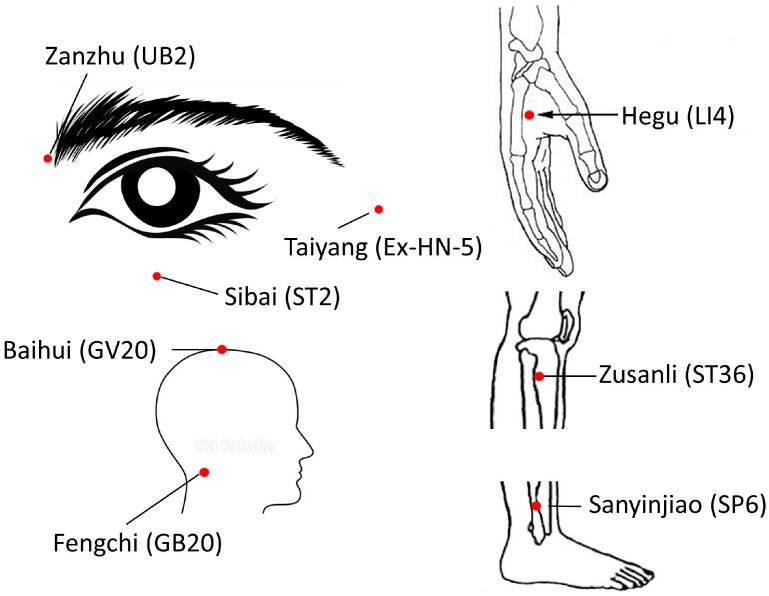
Acupuncture points for group A (acupuncture treatment). UB2 = Urinary Bladder 2; ST2 = Stomach 2; Ex-HN-5 = Extra Point Head/Neck 5; GV20 = Governing Vessel 20; GB20 = Gallbladder 20; LI4 = Large Intestine 4; ST36 = Stomach 36; SP6 = Spleen 6.

All 22 patients in group A received needle acupuncture at traditional point locations (see Figure 1 and Figure 2).

After the patients lay down on a bed in a relaxed manner, the skin at the acupoint locations was disinfected with cotton which contains 75% alcohol. Disposable acupuncture needles (0.25 mm × 25 mm) were used. The needling method used is called mild reinforcing and attenuating, and was performed by inserting the needle at the appropriate depth. The angle of insertion was straight for the points on hands, body, and ankles, and oblique for the points on head, face, and neck. The depth of perpendicular needling was 25 mm, which of oblique is 15 mm. The needles were not stimulated and stayed in the body. After 20 min the needles were removed with dry cotton. An expert panel of acupuncturists from the Tongren Hospital in Beijing, China have reviewed and participated in the selection of the points. Acupuncture was always performed by the same highly experienced acupuncturist.

#### 2.2.2. Moxibustion

**Figure 3:**
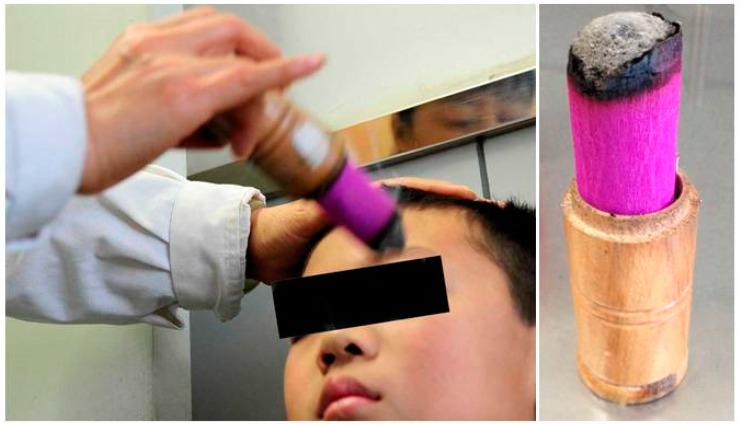
A part of the moxibustion treatment in 22 young patients with myopia (group B).

**Figure 4:**
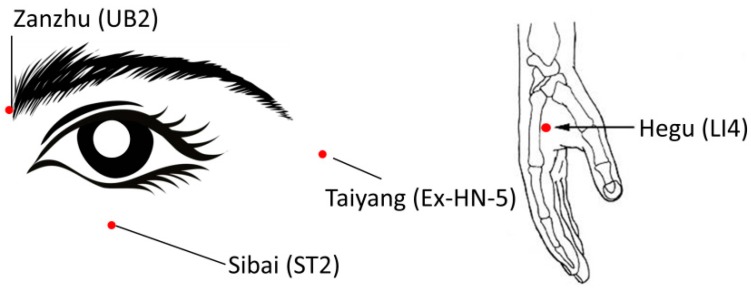
Points for group B (moxibustion treatment). UB2 = Urinary Bladder 2; Ex-HN-5 = Extra Point Head/Neck 5; ST2 = Stomach 2; LI4 = Large Intestine 4.

For moxibustion treatment the patients sat down and closed their eyes. Then moxibustion on the forehead was performed for two minutes moving the stick in a horizontal direction (see Figure 3). Moxibustion was also performed around the eyes for duration of three minutes (moving the moxa in a horizontal direction for one minute, clockwise for one minute, vertical direction for one minute). Then sparrow-pecking Moxa on acupuncture points near the eyes (Ex-HN5, ST2 and UB2) was done for two minutes. This method is characterized by the ignited moxa stick being moved up and down over the point like a bird pecking or moving left and right, or circularly simultaneously. Acupressure was made on the same points simultaneously. In addition, moxibustion around the ears was made for two minutes moving the stick in vertical directions. Sparrow-pecking moxa on LI4 and simultaneous acupressure at the same point for one minute completed the Zhao’s thunder-fire moxa (manufacturer: Chongqing Zhao’s Thunder-fire Moxa Institute of Traditional Medicine, Chongqing, China) procedure. The points for moxibustion are shown in Figure 4.

### 2.3. Evaluation Parameters

Axial lengths were observed before and after treatment with acupuncture or moxibustion. The diopters of the right (OD) and left (OS) eye were evaluated before and after the two treatment methods by a blinded assessor (medical doctor from Tongren Hospital). Compound tropicamide eye drops (0.5% tropicamide, 0.5% phenylephrine hydrochloride) were used before and after the last treatment session.

### 2.4. Statistical Analysis

The statistical analysis was carried out with the computer program SigmaPlot 14.0 (Systat Software, Chicago, IL, USA). Kruskal-Wallis One Way Analysis of Variance (ANOVA) on Ranks for group analysis and box plot analysis was used. In addition, paired t-tests before and after the treatments were carried out. The level of significance was set at *p* < 0.05. The original data can be found at the Department of Traditional Chinese Medicine & Acupuncture, Tongren Hospital affiliated to Capital Medical University, 100730 Beijing, China and at the TCM Research Center Graz, Medical University of Graz, 8036 Graz, Austria.

## 3. Results

All 44 patients finished at least five treatment session (mean sessions: 8.0 ± 1.8 (range: 5–10 treatments)). Due to time reasons, treatment in the children in Beijing was only possible during holiday time. Group analysis of the demographic data showed no statistical difference in age, sex, height, and weight. Acupuncture and moxibustion were accepted well and there were no side effects monitored by the medical doctors of the Tongren Hospital in any of the children. Table 2 demonstrates the mean ± SD of OD and OS from all patients and each from both groups (A and B) respectively.

Altogether myopia was improved (diopters) in 14 eyes of 13 patients (15.9%) with both complementary methods. Using acupuncture, an improvement was observed in seven eyes from six patients out of 22 patients and a similar result (improvement in seven eyes from seven patients out of 22 patients) was noticed in the moxibustion group (group B). However the extent of the improvement was significantly different between the acupuncture (group A: *p* = 0.008) and the moxibustion (group B: No significance).

**Figure 5:**
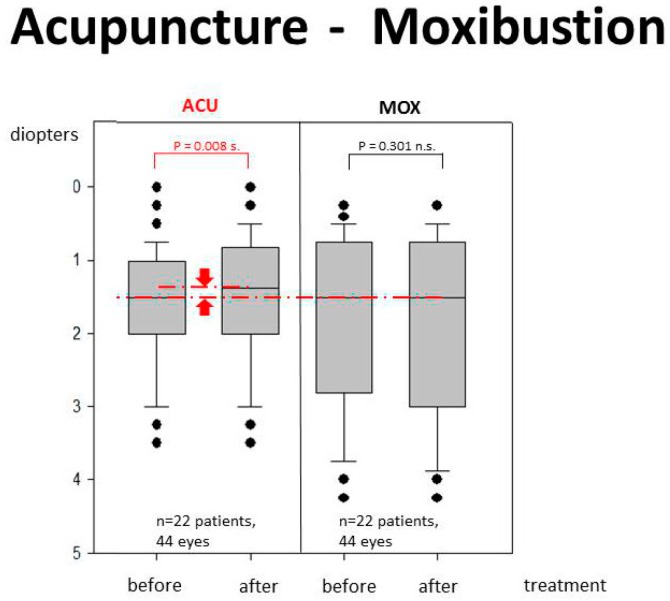
Box-plot of change in diopter values of all 44 subjects before and after treatment. The line in the box indicates the position of the median; the ends of the boxes define the 25th and 75th percentiles. The error bars show the 10th and 90th percentiles, and the dots represent “outliers”. Note the significance (*p* = 0.008 s.) of the decrease in diopters after acupuncture treatment (left part).

The results are graphically presented in Figure 5. The basic values before the treatment of both acupuncture and moxibustion are similar (see median values). Only needle acupuncture treatment changes the diopters in the sense of an improvement of myopic children significantly.

In addition, a group analysis has been performed. The differences in the median values among the two treatment groups (acupuncture and moxibustion) was not great enough to exclude the possibility that the difference is due to random sampling variability; there was not a statistically significant difference (*p* = 0.973 n.s.) between the acupuncture and moxibustion group.

## 4. Discussion

A current literature analysis in the two databases PubMed and Cochrane Library regarding this important topic yielded the following results: up to now (1 June 2018) there are 28,106 published articles in PubMed under the search term “acupuncture” (11,464 at Cochrane Library). The term “myopia” provided 21,252 results in PubMed, and in Cochrane Library 1435 articles. The search term “personal computer” yielded 24,940 results in PubMed, and Cochrane Library 1905 published articles. The search with combined keywords yielded fewer results. The combined search terms “personal computer myopia”, “personal computer acupuncture”, and “acupuncture myopia personal computer” led to no results in Cochrane Library (in PubMed at least double-digit results, excluding “acupuncture myopia personal computer”). The only combined keyword which yielded more results in both databases was “acupuncture myopia”. PubMed listed 46 articles, and in Cochrane Library 22 articles were found.

The result of the database analysis shows that until now there is very little research related to therapeutic aspects of eye diseases and screen handling. Therefore, new research like the present study is absolutely necessary. As already mentioned in the introduction, acupuncture or moxibustion could be possible starting points. The database analysis also clearly shows that acupuncture has already been used in myopia, but currently plays a very minor role in relation to PC-work induced disorders. 

A study from Beijing which has been published in 2015 in PLoS One [2], deals with myopia in high school students. The title of the article is “*Prevalence and associated factors of myopia in high-school students in Beijing*”. The authors came to the worrying result that students aged between 16 and 18 years have an 80% prevalence of myopia. They even speak of a 10% prevalence of severe myopia. When this generation which has grown up with PCs gets older, it will cause enormous costs for the health care system. Experts warn that myopia will win importance as a cause of visual impairment and blindness [2]. 

“*Acupuncture for teenagers with mild to moderate myopia: study protocol for a randomized controlled trial*” is the title of another important work. Especially the safety of acupuncture application in this field and its effectiveness should be investigated for a period of six months. Here is a brief excerpt from the study protocol: This randomized, parallel, blinded clinical trial will be conducted controlled. A total of 100 young people aged between seven and twelve years with mild to moderate myopia are recruited. The patients are randomized into two groups (control group and acupuncture group). Each group consists of 50 young people. In the acupuncture group, the five acupoints Zanzhu bilaterally, Tongziliao, Sibai, Muchuang and Hegu are stimulated daily for nine consecutive days. Then there is a one-day treatment break. Six of these treatment cycles are carried out continuously over a total of 60 days. After six months, a follow-up examination takes place. The primary endpoint is the determination of the diopter [8]. This is in context with our present study and also some acupoint are the same. The study could provide very interesting results. Another work that should be mentioned in the context of this discussion was written by Ming Yeung. According to this work, 3–4% of children are affected by tired eyes. Attention is drawn to a treatment with acupuncture. This method of treatment is according to the authors well tolerated by children and shows promising results [9].

In the following some important experiences from our own research should be mentioned briefly.

Myopia in children and adolescents up to the age of 21 is also well treatable with complementary medical methods. In adults, however, only little success has been recorded [10].

Our study has been performed as a transcontinental research pilot study. This means that two main research teams were involved; one team from Asia (Beijing, China) and one from Europe (Graz, Austria). Although it was a pilot study, the preliminary results are promising. The examinations showed that a total of 14 eyes of 13 patients out of 44 patients showed a significant improvement in myopia. The improvements were better with acupuncture than with moxibustion (comparison before and after treatment). The invasiveness of acupuncture seems to play a role over the non-invasiveness of moxibustion or acupressure.

However, the investigation has also limitations. Firstly, the sample size was very small and in the future sample size will be calculated according to rigorous methodology and considered in the improvement rate of this pilot study. Secondly, the average number of treatment sessions was lower in the moxibustion group than in the acupuncture group. Thirdly, because of ethical principles and actual clinical conditions in China, neither a control group of individuals who did not receive treatment nor a placebo group who received sham acupuncture was included. Fourthly, without a follow-up evaluation it would be difficult to determine if the treatment produces only temporary improvement or more clinically relevant long-term sustainable effects. Nevertheless, the scientific methodological guidelines were followed strictly through the trial, and the outcomes still showed meaningful and effective treatment corresponding to other previous publications. 

## 5. Conclusions

In summary it can be stated that the enormously important topic screen handling, eye diseases, and possible therapeutic aspects with the help of acupuncture or moxibustion has not yet been investigated adequately from a scientific perspective, although some work (mainly from China) already exists. The problem, however, is not only interesting for Asia, also in the Western world ametropia [11] caused by screen handling will increase, as the scientific search and research of this work illustrates.

Our transcontinental pilot study showed that both acupuncture and moxibustion can improve mild and/or moderate myopia of young patients (6–12 years). Acupuncture seems to be more effective than moxibustion in treating myopia however at the moment we do not have conclusive explanations concerning the underling mechanisms of needle and thermal stimulation on myopia from a neurophysiological perspective.

## Figures and Tables

**Table 1:** Demographic data of the 44 patients.

Parameter	Group A	Group B
	Acupuncture (*N* = 22)	Moxibustion (*N* = 22)
Age (years)	9.4 ± 0.9	9.3 ± 1.7
Sex (female, male)	14 f, 8 m	13 f, 9 m
Height (cm)	140.2 ± 6.8	137.2 ± 11.4
Weight (kg)	34.6 ± 8.0	31.8 ± 7.8
Treatment sessions	6.9 ± 1.7	9.1 ± 1.2

**Table 2:** Diopters of the right (OD) and left (OS) eye before and after the two treatment methods (N = number of eyes).

N: Number of Eyes	Both Complementary Treatment Methods	Group A	Group B
		Acupuncture	Moxibustion
OD + OS before	−1.68 ± 1.03 (*N* = 88)	−1.57 ± 0.84 (*N* = 44)	−1.79 ± 1.18 (*N* = 44)
OD + OS after	−1.67 ± 1.08 **^#^** (*N* = 88)	−1.52 ± 0.87 ** (*N* = 44)	−1.81 ± 1.25 (*N* = 44)
OD before	−1.66 ± 1.03 (*N* = 44)	−1.53 ± 0.86 (*N* = 22)	−1.80 ± 1.20 (*N* = 22)
OD after	−1.72 ± 1.08 (*N* = 4)	−1.50 ± 0.88 (*N* = 22)	−1.80 ± 1.20 (*N* = 22)
OS before	−1.69 ± 1.03 (*N* = 44)	−1.60 ± 0.83 (*N* = 22)	−1.81 ± 1.20 (*N* = 22)
OS after	−1.61 ± 1.08 * (*N* = 44)	−1.53 ± 0.87 **^+^** (*N* = 22)	−1.71 ± 1.31 (*N* = 22)

^#^*p* = 0.011; * *p* = 0.002; ** *p* = 0.008; ^+^ *p* = 0.030.
